# The impact and role of hepatic hydrothorax in the prognosis of patients with decompensated cirrhosis: A retrospective propensity score-matched study

**DOI:** 10.3389/fmed.2022.904414

**Published:** 2022-09-06

**Authors:** Bo Ma, Tianling Shang, Jianjie Huang, Zhixin Tu, Yan Wang, Yujin Han, Xiaoyu Wen, Qinglong Jin

**Affiliations:** ^1^Department of Hepatology, The First Hospital of Jilin University, Changchun, Jilin, China; ^2^Department of Neurology, The First Hospital of Jilin University, Changchun, Jilin, China

**Keywords:** hepatic hydrothorax, decompensated cirrhosis, portal hypertension, prognostic, liver disease

## Abstract

**Background and aims:**

Hepatic Hydrothorax (HH) is one of the complications in patients with decompensated cirrhosis and its impact and role in the prognosis of patients with decompensated cirrhosis are not yet clear. Thus, this study aimed to determine the role of HH in patients with decompensated cirrhosis and the long-term impact on their mortality.

**Materials and methods:**

A retrospective study analyzed 624 patients with ascites without pleural effusion in decompensated cirrhosis and 113 patients with HH. Propensity scores were calculated based on eight variables, and the HH and non-HH groups were matched in a 1:1 ratio. The effect and role of HH on the prognosis of patients with decompensated cirrhosis was analyzed using the Kaplan–Meier method and Cox proportional hazards regression model.

**Results:**

A total of 737 patients were included. Out of 113 HH patients, 106 could be matched to 106 non-HH patients. After matching, baseline characteristics were well-balanced. The multifactorial Cox proportional hazards model indicated that hepatic encephalopathy and HH were independent risk factors affecting prognostic survival in patients with decompensated cirrhosis (*P* < 0.01), with risk ratios and 95% confidence intervals (CI) of 2.073 (95% CI: 1.229–3.494, *P* < 0.01) and 4.724 (95% CI: 3.287–6.789, *P* < 0.01), respectively. Prognostic survival was significantly worse in the HH group compared to patients in the non-HH group, with mortality rates of 17.9, 30.1, and 59.4% at 6 months, 1 year, and 2 years in the HH group, compared to 0.9, 3.8, and 5.6% in the non-HH group, respectively. The estimated median survival time was 21 (95% CI: 18–25) months in the HH group and 49 (95% CI: 46–52) months in the non-HH group (*P* < 0.001).

**Conclusion:**

Hepatic hydrothorax is significantly associated with higher mortality in patients with decompensated cirrhosis and is a highly negligible independent decompensated event affecting their prognosis.

## Introduction

Hepatic hydrothorax (HH) is an important decompensated event in cirrhosis that needs further study (1). In individuals with a chronic liver illness and portal hypertension but no underlying cardiac disease, HH is often described as a leaky pleural effusion that is typically more than 500 mL (2). HH, whose incidence is approximately 5–15% of cases, is a rare complication of end-stage liver disease that can lead to hypoxia, respiratory distress, and infection. HH is predictive of a poor prognosis, and its occurrence is not associated with a specific cause of cirrhosis (3, 4). Most research points to the creation of peritoneal-pleural defects as the pathophysiological mechanism by which HH occurs (5–11). These peritoneal-pleural flaws can be either microscopic or macroscopic, depending on the size of the defect (5–11). Furthermore, these abnormalities are more prevalent on the right side of the diaphragm, which is more fibrous and prone to collagen fiber degradation; this is a significant reason for the prevalence of right-sided pleural effusions in patients with HH (1). Decompensated cirrhosis is often combined with a variety of complications, including ascites, hepatic encephalopathy, liver failure, spontaneous peritonitis, gastrointestinal bleeding, and hepatorenal syndrome; however, few clinicians have focused on and explored HH (12, 13). Although HH usually occurs in advanced liver disease, the impact and role of HH in the prognosis of patients with decompensated cirrhosis is currently unknown. Therefore, this study aimed to determine the role of HH plays in patients with decompensated cirrhosis and the long-term impact on their mortality.

## Materials and methods

### Study population

**FIGURE 1:**
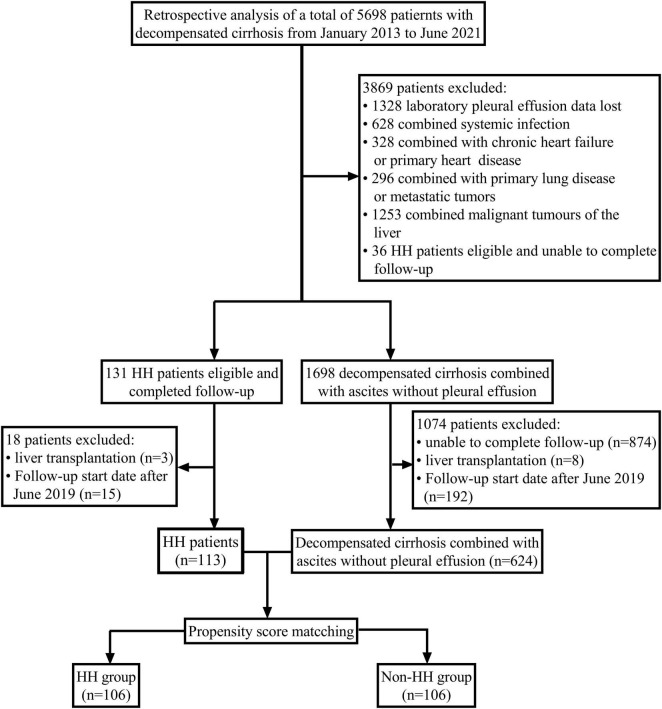
Study flow diagram. HH, hepatic hydrothorax.

We performed a retrospective cohort analysis on 5,698 patients diagnosed with decompensated cirrhosis at the Department of Hepatology, First Hospital of Jilin University, China, from January 2013 to June 2021. A total of 624 patients with decompensated cirrhosis with ascites without pleural effusion and 113 patients with HH were further screened, and patients were divided into the HH group (*n* = 106) and non-HH group (*n* = 106) using propensity score matching, and follow-up data were collected until December 30, 2021. We defined patients with decompensated cirrhosis with hepatic pleural fluid as the case group and patients with decompensated cirrhosis without hepatic pleural fluid as the control group. All relevant clinical and laboratory data at the time of first diagnostic admission, including a complete medical history, were collected. Decompensated cirrhosis and decompensating events have been defined according to the most recent EASL Clinical Practice Guidelines on decompensated cirrhosis (14). Inclusion criteria were: (1) cirrhosis diagnosis, either proven by biopsy or based on clinical symptoms in the clinic which were consistent with cirrhosis; (2) the presence of ascites or pleural effusion on chest radiograph, lung computed tomography (CT), and abdominal CT, the presence of ascites or pleural effusion can be seen; (3) pleural effusion compatible with the recognized characteristics of hepatic pleural fluid, but not consistent with the presence of infection, cancer, or other known chronic illness; (4) no history of cardiopulmonary disease, including congestive heart failure; and (5) the presence of esophageal varices, portal hypertensive gastropathy, ascites, and an increased hepatic venous pressure gradient, all of which are indicators of portal hypertension. The subject exclusion criteria were as follows: (1) patients with multiple malignancies or significant organ failure; (2) patients on hormonal or immunosuppressive medications; and (3) patients with insufficient clinical data and ancillary test information. On admission, ultrasonography was used to detect ascites and pleural effusion in all patients with decompensated cirrhosis. Additionally, all patients with decompensated cirrhosis underwent conventional chest radiographs or lung CT scans to detect any underlying lung illness (pneumonia, tumors, and other lesions). Cardiovascular ultrasonography was performed on patients who had a clinical, biochemical, or electrocardiographic suspicion of heart failure in order to rule out decompensated heart disease. Finally, 212 patients with HH (*n* = 106) and non-HH patients (*n* = 106) with a follow-up period greater than or equal to 2 years were included in this analysis (Figure 1). The study procedure was approved by the Ethics Committee of Jilin University’s First Hospital and was carried out in accordance with the principles of the Helsinki Declaration. All participants provided written informed consent.

### Study variables

All demographic and clinical information, including age and gender, was confirmed directly from the electronic medical record, including demographic data, serum biochemical information [alanine aminotransferase (ALT), aspartate aminotransferase (AST), Alkaline phosphatase (ALP), gamma-glutamyl transpeptidase (GGT), total bilirubin (TBIL), albumin (ALB), Creatinine (Cr), serum sodium, International normalized ratio (INR), neutrophil-to-lymphocyte ratio (NLR), prothrombin time (PT)], and imaging features. The diagnosis of cirrhosis was confirmed by liver biopsy or ultrasound without regard to the presence of portal hypertension. The Child–Pugh score was computed based on the original publication by Pugh et al. (15). The model for end-stage liver disease (MELD)-Na score was computed based on the report of Biggins et al. (16). The albumin-bilirubin (ALBI) scoring and grading system was employed in this study exactly as outlined by Johnson et al. (17).

The applicable formula is as follows: ALBI score = −0.085 × (ALB, g/L) + 0.66 × lg (TBIL, μmol/L). Grade 1: ALBI score ≤ −2.6; Grade 2: −2.6 < ALBI score ≤ −1.39; Grade 3: ALBI score > −1.39. MELD score = 3.78 × In (TBIL, μmol/L) + 11.2 × In (INR) + 9.57 × In (Cr, μmol/L) + 6.4 × Etiology (0 for biliary or alcoholic cirrhosis, 1 for other causes). MELD-Na score = MELD + 1.59 × (135-Na, mmol/L), where when serum Na ≥ 135 mmol/L is calculated as 135 mmol/L, Na < 120 mmol/L is calculated as 120 mmol/L, and 120–135 mmol/L is calculated according to the specific value. The Child–Pugh score is based on five indicators: serum ALB, TBIL, PT, ascites, and hepatic encephalopathy, with a grade A score of 5–6, a grade B score of 7–9, and a grade C score of 10–15. The evaluation criteria for the volume of pleural effusion are as follows: If the quantity of pleural fluid measured by ultrasonography or chest X-ray is larger than 6 cm or over the seventh rib space, it is a significant amount; if the fluid level is between 3 and 6 cm or over the eighth intercostal gap, it is a moderate quantity; if it is less than 3 cm or the rib-diaphragm angle is blunt, it is a little amount. Hepatic encephalopathy grades 2 to 4 were used to define overt encephalopathy in accordance with the West Haven criteria (18). In addition, ascites was categorized according to the most recent position document produced by the International Ascites Club (19).

All blood samples were analyzed in the clinical laboratory of Jilin University’s First Hospital. An automated biochemical analyzer identified the blood biochemistry indicators (7600–210, Hitachi, Japan). Complete blood count was measured using an SYSMEX XN-9000 hematology analyzer (Sysmex Corporation, Kobe, Japan) according to the manufacturer’s instructions. The coagulation tests were performed by the clotting method on the automatic coagulometer “SYSMEX CS-5100” (Sysmex Corporation, Kobe, Japan).

### Outcomes

We collected treatment details, date of death, liver transplantation, and final follow-up for HH and non-HH patients. Decompensation of cirrhosis was defined as the development of liver-related complications such as variceal bleeding, ascites, spontaneous bacterial peritonitis, hyponatremia, liver failure, or hepatic encephalopathy. Outcomes were death or loss of visit as endpoint events. We determined the association of various factors with death at the primary time point analysis, which was calculated from the start date of diagnosis in HH and non-HH patients to the date of death or the last follow-up.

### Statistical analysis

Categorical data are expressed as numbers (n) and proportions (%). Continuous variables are expressed as means (standard deviation) for regularly distributed data, and non-normally distributed data are reported as medians (quartile 25–quartile 75). The Shapiro–Wilk prevailed for the normality test. In addition, the Schoenfeld residuals for continuous variables and the graphical technique for categorical data were used to evaluate the proportional hazard assumption. The variables that appeared to fulfill the proportional hazard assumption were included in the univariate Cox proportional hazard model to evaluate the impact of various factors on survival time. The risk ratio and its 95% confidence interval (CI) were used to estimate the contribution of each variable. The multivariate Cox proportional hazard model consisted of all factors that significantly impacted survival (*P* < 0.1). The factors that may be considered were gradually eliminated from the multivariate Cox regression model, and the change in the hazard ratio value of the target variable (the first variable) was noted when a variable was eliminated. The variable was eventually retained if the change in the hazard ratio value following elimination was more than 10%. Otherwise, the variable was eliminated. The Cox regression models and the Kaplan–Meier method of log-rank tests were used to compare the survival outcomes for categorical variables. The Kaplan–Meier estimates were utilized to show survival curves. Hazard ratios and their related 95% CIs were the outcomes of the Cox proportional hazards regression analysis. Our investigation into the causes of death and survival was exploratory. All statistical analyses were performed using SPSS Statistics 26 (IBM, New York, NY, United States) and R version 4.1.3. A two-sided *P*-value < 0.05 was considered statistically significant.

### Propensity score matching

Propensity score matching (PSM) was performed to avoid confounding by indication and was reported in accordance with the guidelines of Lonjon et al. (20). It was performed using SPSS Statistics 26 (IBM, New York, NY, United States). A logistic regression model was used to calculate the propensity scores. The authors considered all relevant variables and agreed on which variables to include in the model. The final model included the following variables: age, cholinesterase (CHE), ALB, prothrombin activity (PTA), Cr, MELD score, MELD-Na score, and the ALBI score. Based on this propensity score, the HH group was matched 1:1 with the non-HH group using a caliper width of 0.2. To evaluate the balance, the standardized mean differences for each baseline variable were computed before and after matching. The ideal equilibrium was defined as a standardized mean difference between 0.1 or 0.1 and 0.

## Results

### Comparison of baseline clinical characteristics of patients in the original group

A total of 624 patients with no HH and 113 patients with HH were compared for baseline clinical characteristics, with 84.7% of patients in the non-HH group and 15.3% in the HH group. Of the 737 patients, 484 were males and 253 were females, with a mean age of 55.65 ± 11.91 years. Among them, there were 326 patients with hepatitis B cirrhosis, 81 with hepatitis C cirrhosis, 110 with alcoholic cirrhosis, 42 with primary biliary cirrhosis and 178 with cirrhosis of unknown origin. There were no statistically significant differences between the two groups in terms of gender, age, etiology, AST, ALT, ALP, TBIL, direct bilirubin, indirect bilirubin, PT, INR, Child–Pugh classification, Child–Pugh score, presence of hepatic encephalopathy, renal insufficiency, liver failure, gastrointestinal bleeding, hyponatremia, and spontaneous peritonitis (*P* > 0.05). Liver function was worse in the HH group, with GGT, CHE, ALB, and PTA significantly lower than those in the non-HH group, all with statistically significant differences (*P* < 0.05). Fasting blood glucose, blood urea nitrogen, Cr, MELD score, MELD-Na score, and ALBI score were significantly higher in the HH group than in the non-HH group, all with statistically significant differences (*P* < 0.05). Compared to the non-HH group, patients in the HH group had a more severe degree of ascites and ALBI grading (*P* < 0.01). In addition, short and long-term mortality rates were significantly higher in the HH group than in the non-HH group, with 6-month and 2-year mortality rates of (1.3% vs. 18.6%, *P* < 0.01) and (4.2% vs. 58.4%, *P* < 0.01), respectively (see Table 1).

**TABLE 1:** Comparison of baseline information between patients in the original and propensity score matching (PSM) groups.

Variable	Original group		PSM group		*P-*value	
	Non-HH group	HH group	Non-HH group	HH group	Original group	PSM group
N	624 (84.7%)	113 (15.3%)	106 (50%)	106 (50%)		
Female (N, %)	209 (33.5%)	44 (38.9%)	35 (33%)	41 (38.7%)	0.262	0.390
Age (years)	55.54 ± 11.86	56.25 ± 12.19	56.15 ± 12.24	55.92 ± 12.32	0.532	0.797
Etiology (N, %)					0.202	0.896
Hepatitis B virus (HBV)	279 (44.7%)	47 (41.6%)	48 (59.4%)	44 (41.5%)		
Hepatitis C virus (HCV)	68 (10.9%)	13 (11.5%)	10 (16%)	11 (10.4%)		
Alcohol	86 (13.8%)	24 (21.2%)	20 (8.5%)	24 (22.6%)		
Primary biliary cirrhosis (PBC)	34 (5.4%)	8 (7.1%)	6 (2.8%)	8 (7.5%)		
Other	157 (25.2%)	21 (18.6%)	22 (13.2%)	19 (17.9%)		
AST (U/L)	46.00 (31.05–77.22)	44.90 (33.00–69.85)	50.05 (35.22–70.62)	44.60 (32.67–70.82)	0.858	0.359
ALT (U/L)	28.55 (19.05–50.37)	30.20 (20.60–50.00)	32.00 (20.80–48.62)	29.60 (19.10–50.75)	0.698	0.837
GGT (U/L)	51.00 (25.80–122.92)	38.30 (20.00–81.00)	55.90 (25.87–100.92)	35.00 (19.90–80.50)	0.014	0.048
ALP (U/L)	99.90 (72.87–138.65)	94.50 (71.75–140.60)	100.85 (78.62–155.55)	90.75 (71.45–134.07)	0.993	0.162
CHE (U/L)	2577.0 (1965.7–3530.0)	1989.0 (1540.5–2790.0)	1907.0 (1431.7–2667.2)	1988.5 (1547.2–2772.5)	<°0.01	0.336
TBIL (umol/L)	34.75 (18.92–77.97)	44.70 (23.95–80.80)	44.40 (26.60–86.85)	44.00 (24.07–79.30)	0.150	0.599
DBIL (umol/L)	13.25 (7.00–38.30)	15.80 (9.55–36.55)	19.85 (10.82–37.67)	16.40 (9.57–37.70)	0.294	0.430
IBIL (umol/L)	19.50 (10.82–40.22)	25.00 (14.65–44.30)	24.25 (14.52–43.90)	24.85 (14.67–43.95)	0.073	0.768
ALB (g/L)	28.39 ± 6.34	26.01 ± 5.05	26.35 ± 5.28	26.09 ± 5.03	0.021	0.058
FBG (mmol/L)	5.71 (4.78–7.36)	6.39 (4.98–7.91)	6.29 (5.12–8.67)	6.38 (4.94–7.81)	<°0.01	0.851
BUN (mmol/L)	5.64 (4.31–7.90)	6.33 (4.73–9.69)	6.01 (4.44–9.55)	6.19 (4.71–9.83)	<°0.01	0.359
PT (s)	15.80 (13.70–19.77)	16.10 (13.90–19.55)	16.30 (13.67–18.42)	16.25 (13.82–19.77)	0.976	0.741
PTA (%)	64.00 (47.00–74.00)	55.00 (42.50–70.00)	58.19 ± 19.43	58.11 ± 22.35	0.017	0.830
INR	1.32 (1.18–1.59)	1.36 (1.19–1.61)	1.37 (1.19–1.59)	1.36 (1.18–1.63)	0.528	0.921
Child-Pugh grade					0.307	1.00
Child-Pugh grade A (N, %)	4 (0.6%)	2 (1.8%)	1 (0.9%)	1 (0.9%)		
Child-Pugh grade B (N, %)	218 (34.9%)	34 (30.1%)	32 (30.2%)	32 (30.2%)		
Child-Pugh grade C (N, %)	402 (64.4%)	77 (68.1%)	73 (68.9%)	73 (68.9%)		
Child-Pugh score	10.45 ± 1.96	10.56 ± 1.99	11.00 (9.00–12.00)	10.50 (9.00–12.00)	0.898	0.860
MELD score	7.35 (3.18–12.67)	10.00 (5.43–14.27)	10.74 ± 6.95	10.53 ± 6.79	<°0.01	0.637
MELD-Na score	5.47 (−1.06–13.83)	9.26 (1.96–16.50)	10.02 (2.07–17.99)	9.24 (12.12–15.65)	<°0.01	0.554
ALBI score	−1.33 ± 0.68	−1.12 ± 0.54	−1.12 ± 0.55	−1.12 ± 0.53	0.016	0.227
ALBI grade					<°0.01	0.385
ALBI grade 1 (N, %)	12 (1.9%)	2 (1.8%)	5 (4.7%)	2 (1.9%)		
ALBI grade 2 (N, %)	277 (44.4%)	28 (24.8%)	29 (27.3%)	25 (23.6%)		
ALBI grade 3 (N, %)	335 (53.7%)	83 (73.5%)	72 (67.9%)	79 (74.5%)		
Cr (umol/L)	66.00 (53.72–79.45)	70.90 (57.80–90.00)	73.45 (54.37–88.25)	69.95 (57.30–92.72)	0.042	0.936
6 months mortality (N, %)	8 (1.3%)	21 (18.6%)	1 (0.9%)	19 (17.9%)	<°0.01	<°0.01
2 years mortality (N, %)	26 (4.2%)	66 (58.4%)	6 (5.6%)	63 (59.4%)	<°0.01	<°0.01
Ascites grade					<°0.01	0.130
Grade 1 ascites (N, %)	123 (19.7%)	7 (6.2%)	11 (10.3%)	5 (4.7%)		
Grade 2 ascites (N, %)	46 (7.4%)	18 (15.9%)	23 (21.6%)	17 (16%)		
Grade 3 ascites (N, %)	455 (72.9%)	88 (77.9%)	72 (67.9%)	84 (79.2%)		
Hepatic encephalopathy (N, %)	83 (13.3%)	14 (12.4%)	13 (12.3%)	14 (13.2%)	0.792	0.837
Renal insufficiency (N, %)	109 (17.5%)	22 (19.5%)	31 (29.2%)	22 (20.8%)	0.609	0.153
Liver failure (N, %)	105 (16.8%)	17 (15.0%)	15 (14.2%)	16 (15.1%)	0.639	0.846
Gastrointestinal bleeding (N, %)	91 (14.6%)	16 (14.2%)	13 (12.3%)	16 (15.1%)	0.906	0.549
Hyponatremia (N, %)	238 (38.1%)	44 (38.9%)	54 (50.9%)	41 (38.7%)	0.778	0.073
Spontaneous peritonitis (N, %)	121 (19.4%)	15 (13.3%)	31 (29.2%)	29 (%)	0.107	0.760

Data are expressed as mean (± standard deviation), median (quartile 25, quartile 75), or number (proportion). ALT, alanine aminotransferase; AST, aspartate aminotransferase; ALP, alkaline phosphatase; GGT, gamma-glutamyl transpeptidase; CHE, cholinesterase; ALB, albumin; TBIL, total bilirubin; DBIL, direct bilirubin; IBIL, indirect bilirubin; Cr, Creatinine; BUN, blood urea nitrogen; FBG, fasting blood glucose; INR, International normalized ratio; PT, prothrombin time; PTA, Prothrombin activity; MELD, model for end-stage liver disease; ALBI, Albumin-bilirubin; PSM, propensity score-matched; HH, Hepatic Hydrothorax.

### Comparison of baseline clinical characteristics of hepatic hydrothorax and non-hepatic hydrothorax patients after propensity score matching

In our effort to more directly assess the impact of HH on the prognosis of patients with decompensated cirrhosis, eligible HH patients (624) were matched with non-HH patients (113) with regard to age, CHE, ALB, PTA, Cr, MELD score, MELD-Na score, and ALBI score. They were propensity score-matched, with a total of 106 pairs being matched. After matching, a total of 106 pairs were matched between the two groups in the presence or absence of hepatic encephalopathy, renal insufficiency, liver failure, gastrointestinal bleeding, hyponatremia, spontaneous peritonitis, sex, age, etiology, AST, ALT, ALP, CHE, ALB, direct bilirubin, indirect bilirubin, fasting blood glucose, blood urea nitrogen, Cr, PT, PTA, INR, Child–Pugh score, MELD score, MELD–Na score, ALBI score, Child-Pugh grade, ALBI grading, and ascites severity which did not show statistically significant differences (*P* > 0.05). GGT was significantly lower in the HH group than in the non-HH group, with statistically significant differences (*P* < 0.05). In addition, there was a greater intimal width of the portal vein in the HH group than in the non-HH group, with a statistically significant difference (*P* < 0.05). Finally, short- and long-term mortality rates were significantly higher in the matched HH group than in the non-HH group, with mortality rates at 6 months and 2 years of (0.9% vs. 17.9%, *P* < 0.01) and (5.6% vs. 59.4%, *P* < 0.01), respectively (see Table 1).

### Prognostic impact and role of hepatic hydrothorax after propensity score matching in patients with decompensated liver cirrhosis

Prognostic-related factors were analyzed for 212 patients without transplantation after PSM, including 106 in the HH group and 106 in the non-HH group. Univariate survival analysis using Cox proportional risk model for the matched group (including HH and non-HH patients) showed that hepatic encephalopathy, HH, TBIL, PT, MELD score, MELD-Na score, and ALBI score were associated with prognostic survival (*P* < 0.05). Hepatic encephalopathy, HH, and PT were significantly associated with prognostic survival (*P* < 0.01), with HH having a significant effect on mortality with a risk ratio and 95% CI of 3.981 (95% CI: 2.867–5.528, *P* < 0.01). Factors associated with survival (*P* < 0.1) in the univariate Cox proportional risk model for the whole cohort after matching, such as liver failure, hepatic encephalopathy, HH, TBIL, PT, MELD score, MELD-Na score, C-P score, ALBI score, and ALBI grade 3 were included in the multivariate Cox proportional risk model, indicating that hepatic encephalopathy and HH were independent risk factors affecting the prognostic survival of patients with decompensated cirrhosis independent risk factors for prognostic survival with risk ratios and 95% CIs of 2.073 (95% CI: 1.229–3.494, *P* < 0.01) and 4.724 (95% CI: 3.287–6.789, *P* < 0.01), respectively (see Table 2).

**TABLE 2:** Univariate and multivariate Cox proportional hazards model in the entire matched cohort (*n* = 212 patients).

Variables	Univariate analysis		Multivariate analysis	
	HR (95% CI)	*P-*value	HR (95% CI)	*P-*value
Age (years)	0.990 (0.997, 1.003)	0.126		
Male	1.160 (0.851, 1.581)	0.348		
Liver failure	1.565 (0.979, 2.501)	0.061	1.303 (0.724, 2.344)	0.377
Hepatic encephalopathy	1.873 (1.199, 2.924)	<°0.01	2.104 (1.321, 3.349)	0.002
Digestive bleeding	1.365 (0.891, 2.093)	0.153		
Spontaneous peritonitis	1.251 (0.868, 1.803)	0.230		
Hyponatremia	1.166 (0.865, 1.572)	0.312		
Renal insufficiency	1.084 (0.766, 1.533)	0.651		
Hepatic hydrothorax	3.981 (2.867, 5.528)	<°0.01	4.271 (3.048, 5.987)	<°0.001
Grade III ascites	0.882 (0.579, 1.342)	0.556		
AST (U/L)	1.000 (0.997, 1.003)	0.994		
ALT (U/L)	1.001 (0.996, 1.005)	0.783		
GGT (U/L)	1.000 (0.998, 1.001)	0.788		
ALP (U/L)	0.999 (0.997, 1.001)	0.437		
ALB (g/L)	0.976 (0.947, 1.007)	0.124		
TBIL (umol/L)	1.002 (1.000, 1.004)	0.047	1.001 (0.999, 1.003)	0.367
PT (s)	1.046 (1.012, 1.082)	<°0.01	1.016 (0.974, 1.060)	0.457

M, male; F, female; ALT, alanine aminotransferase; AST, aspartate aminotransferase; ALP, alkaline phosphatase; GGT, gamma-glutamyl transpeptidase; TBIL, total bilirubin; ALB, albumin; PLT, platelet; TT, Thrombin time; PT, prothrombin time; HH, Hepatic Hydrothorax; HR, hazard ratio; CI, confidence interval.

### The Kaplan–Meier graft-free survival curves for the hepatic hydrothorax and non-hepatic hydrothorax groups after propensity score matching

**FIGURE 2:**
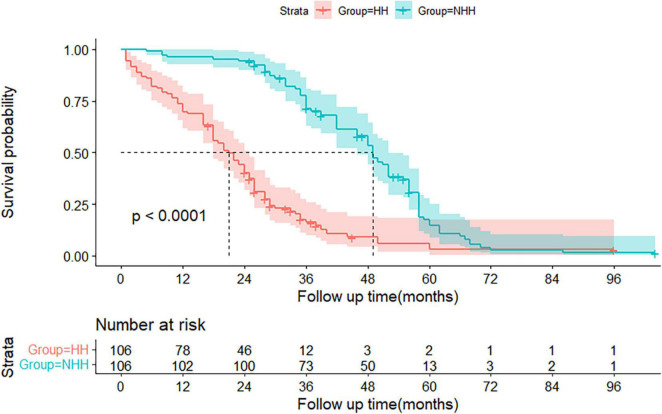
The Kaplan-Meier graft-free survival curves for the hepatic hydrothorax (HH) and non-HH groups. The 95% confidence interval of the survival probability is marked by the shading. The dotted line indicates the median overall survival. The displayed *p* values follow from the log-rank test. Every tick mark indicates a censored patient. HH, hepatic hydrothorax.

Based on these data, the entire matched cohort was analyzed by subgroup based on the presence or absence of HH. Furthermore, prognostic survival was significantly worse in the HH group than in the non-HH group, with mortality rates of 17.9, 30.1, and 59.4% in the HH group compared to 0.9, 3.8, and 5.6% in the non-HH group, at 6 months, 1°year, and 2 years, respectively. The estimated median survival time was 21 (95% CI: 18–25) months in the HH group and 49 (95% CI: 46–52) months in the non-HH group (*P* < 0.0001) (see Figure 2).

## Discussion

We conducted this retrospective cohort study to describe the long-term survival of patients with decompensated cirrhosis at a large tertiary care center in northeast China and to determine the prognostic impact and role of HH as a specific complication. Our results show that HH has a significantly higher graft-free mortality rate in patients with decompensated cirrhosis, both in the short and long term. In other words, the probability of surviving transplant-free was significantly lower in the HH group than in the non-HH group. Furthermore, HH was independently associated with prognostic survival in patients with decompensated cirrhosis. These findings reveal the role of HH in the prognosis of patients with decompensated cirrhosis, allowing for early identification of high-risk patients and providing important insights into the early management of patients with HH in decompensated cirrhosis.

Our data study showed that patients with HH with propensity score-matched indicators of liver function, coagulation, and renal function had significantly higher long-term mortality than patients with ascites alone without HH. Overall, most patients with HH were at the more extreme end of the severity spectrum of decompensated cirrhosis, with significantly lower GGT, CHE, serum ALB, and PTA compared to the non-HH group. In addition, they had significantly higher serum fasting glucose, urea nitrogen, serum Cr, MELD score, MELD-Na score, and ALBI score than the non-HH group. There is now general agreement on the impact of the MELD score, hepatic encephalopathy, hyponatremia, and indicators such as ALB, bilirubin, and renal function on the overall poor prognosis of patients with decompensated cirrhosis (21–25). However, the unique aspect of our study is to further clarify the role of HH as a predictor of long-term mortality. Several studies have shown that patients with hepatic pleural fluid have poorer prognoses (26–28), although recent reports have compared this with relevant patient groups (29). These data are the basis for the current recommendations to refer patients with cirrhosis with ascites and hepatic pleural fluid for liver transplantation or transjugular intrahepatic portosystemic shunt treatment (30–33). It has also been shown that patients with hepatic pleural fluid have a four-fold higher mortality rate, further confirming that hepatic pleural fluid is a manifestation of impending liver failure (34). In a recent study (29), the risk of ACLF and inpatient mortality was more than doubled in patients with HH, requiring therapeutic thoracentesis compared to patients hospitalized with sclerosis.

There are few studies on the mortality of patients with decompensated cirrhosis and HH, especially short and medium-term mortality. Further, data from prospective sources are limited. In a retrospective study of 77 patients with HH (35), their reported 30-day, 90-day, and 1-year mortality rates were 10, 26, and 57%, respectively. In a large retrospective study published in Taiwan in 2018 (28), a total of 3,487 patients with cirrhosis combined with pleural effusion requiring drainage were included, and their 30-day, 90-day, 1-year, and 3-year mortality rates were 20.1, 40.2, 59.1, and 75.9%, respectively. In patients with cirrhosis, the presence of pleural effusion predicts a poor long-term prognosis. In a recent retrospective study of 100 patients with HH (36), their 30-day and 6-month mortality rates were 19 and 32%, respectively. In this study, 101 patients with known clinical endpoint events had mortality rates of 8.9, 15.8, 22.8, and 37.6% at 30 days, 90 days, 6 months, and 1°year, respectively, which are in the middle of the range of these data.

Hepatic hydrothorax is a unique complication in patients with decompensated cirrhosis. Few previous studies have grouped patients by the presence or absence of HH and thus matched them for propensity scores, thus exploring survival differences between the two groups. In a study by Matei et al. (36) which used a similar approach to ours, a multifactorial Cox proportional risk model showed that HH, MELD-Na score, ALBI classification, hepatorenal syndrome, and grade III ascites remained significantly different in the entire cohort of 194 patients after matching (*P* < 0.05). Contrastingly, our data was based on 212 patients after matching, and the multifactorial Cox proportional risk model showed that HH and hepatic encephalopathy were independent risk factors for prognostic survival in patients with decompensated cirrhosis. Our findings are consistent with those of Matei et al. (36), showing that HH is an independent risk factor for prognostic survival in patients with decompensated cirrhosis, which strongly suggests that HH is an easily overlooked decompensated event. Although the impact of HH on direct prognosis is relatively small compared to other previously known common decompensated events (ascites, renal insufficiency, hepatorenal syndrome, hepatic encephalopathy, and ruptured esophageal variceal hemorrhage), HH appears to be considered by the clinician at a later point in the timeline of decompensated disease progression, thereby significantly altering prognosis further. Therefore, early identification of HH in patients with decompensated cirrhosis and timely diagnosis and, consequently, timely management of patients with a combination of other decompensated events is important as HH can be a marker manifestation of poor prognosis.

Our study had several limitations. First, because this was a single-center and retrospective study, there may have been systematic bias in the selection of inclusion and exclusion criteria, excluding the influence of subjective factors by patients and medical record-keepers at the time of our data collection. In addition, for non-HH patients, the majority were excluded due to the “inability to complete follow-up,” which may have contributed to the existence of survival bias in the cohort. Second, although we adjusted for confounding factors, residual confounding is still possible. Third, the number of patients who underwent transjugular intrahepatic portosystemic shunt and liver transplantation was too small to be included in the Cox proportional risk model survival analysis. Fourth, many complications could be treated and are not life-threatening with recent treatment procedures. In this regard, this cohort recruited patients for 9 years from 2013 to 2021. During this period, therapeutic strategies for these decompensated events might have improved and thereby could have produced a bias.

In summary, our findings suggest that HH is an independent risk factor for poor prognostic survival in patients with decompensated cirrhosis. However, properly designed prospective studies are required for further confirmation.

## Data availability statement

The raw data supporting the conclusions of this article will be made available by the authors, without undue reservation.

## Ethics statement

The studies involving human participants were reviewed and approved by the First Hospital of Jilin University. The patients/participants provided their written informed consent to participate in this study.

## Author contributions

JH, ZT, YW, and YH collected the data and interpreted the diagnosis. BM analyzed the literature and wrote the manuscript. TS helped in collecting the data and edited the manuscript. XW and QJ provided framework for the study, reviewed, and edited the manuscript. All authors have read and agreed to the final version of the manuscript.

## Abbreviations

M: male
F: female
ALT: alanine aminotransferase
AST: aspartate aminotransferase
ALP: alkaline phosphatase
GGT: gamma-glutamyl transpeptidase
CHE: cholinesterase
ALB: albumin
TBIL: total bilirubin
DBIL: direct bilirubin
IBIL: indirect bilirubin
Cr: creatinine
BUN: blood urea nitrogen
FBG: fasting blood glucose
NLR: neutrophil-to-lymphocyte ratio
PLT: platelet
TT: thrombin time
INR: international normalized ratio
PT: prothrombin time
PTA: prothrombin activity
HRS: hepatorenal syndrome
TIPS: transjugular intrahepatic portosystemic shunt
LT: liver transplantation
MELD: model for end-stage liver disease
ALBI: albumin-bilirubin
CT: computed tomography
PSM: propensity score matching
HH: hepatic hydrothorax
HR: hazard ratio
CI: confidence interval.
